# Efficient synthesis and replication of diverse sequence libraries composed of biostable nucleic acid analogues†

**DOI:** 10.1039/d2cb00035k

**Published:** 2022-08-30

**Authors:** John R. D. Hervey, Niklas Freund, Gillian Houlihan, Gurpreet Dhaliwal, Philipp Holliger, Alexander I. Taylor

**Affiliations:** Cambridge Institute of Therapeutic Immunology & Infectious Disease (CITIID), University of Cambridge Cambridge CB2 0AW UK ait29@cam.ac.uk; Medical Research Council Laboratory of Molecular Biology Cambridge CB2 0QH UK ph1@mrc-lmb.cam.ac.uk

## Abstract

Functional nucleic acids can be evolved *in vitro* using cycles of selection and amplification, starting from diverse-sequence libraries, which are typically restricted to natural or partially-modified polymer chemistries. Here, we describe the efficient DNA-templated synthesis and reverse transcription of libraries entirely composed of serum nuclease resistant alternative nucleic acid chemistries validated in nucleic acid therapeutics; locked nucleic acid (LNA), 2′-*O*-methyl-RNA (2′OMe-RNA), or mixtures of the two. We evaluate yield and diversity of synthesised libraries and measure the aggregate error rate of a selection cycle. We find that in addition to pure 2′-*O*-methyl-RNA and LNA, several 2′OMe-RNA/LNA blends seem suitable and promising for discovery of biostable functional nucleic acids for biomedical applications.

## Abstract 2

Blends of engineered polymerases enable efficient DNA-templated synthesis and reverse transcription of diverse-sequence oligonucleotide libraries composed of locked nucleic acid (LNA), 2′-*O*-methyl-RNA (2′OMe-RNA), or mixtures of the two.

## Graphical abstract

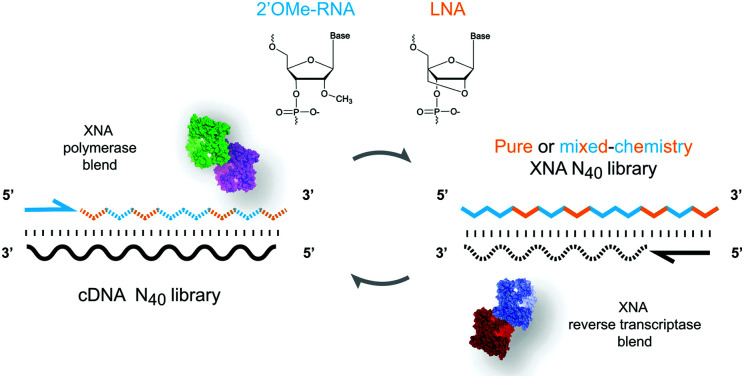

## Introduction

Single-stranded nucleic acids can adopt sophisticated 3D structures, enabling functions as “chemical antibodies” (aptamers) and catalysts (ribo-, DNA- and XNAzymes), which form the basis of an array of biomedical technologies with potential applications in diagnosis and therapy.^1–6^ As design of such functional sequences (beyond antisense reagents) *ab initio* is not yet possible, they must be discovered through *in vitro* selection and evolution, which typically involves cycles of synthesis, panning and recovery of sequence-diverse libraries (containing up to 10^15^ variants), formalised as “systematic evolution ligands by exponential enrichment (SELEX)”.^7,8^

For applications *in vivo*, or in the presence of biological fluids, modified or non-natural nucleic acids (also known as xeno nucleic acids, XNAs) are generally advantageous over DNA or RNA due to improved nuclease resistance inherent in alternative backbone chemistries with modified sugars and congeners.^9–11^ Although post-SELEX modification of functional oligonucleotides with analogues is possible, this can reduce or abolish function and precludes the selection of novel XNA structures stable under physiological conditions. Instead, substitution of one or two of the four RNA or DNA nucleotides with analogues such as 2′-fluoro- and 2′-aminopyrimidines has been used to prepare partially-modified libraries for “modSELEX”,^12,13^ yielding mixed-chemistry aptamers with improved biostability. However, such mixed chemistry aptamers (although generally more stable than DNA or RNA alone) remain vulnerable to nuclease degradation due to the presence of unmodified segments.

By comparison, comparatively few examples of “X-SELEX”^14,15^ selections involving complete substitution of all four nucleoside triphosphates ((d)NTPs) with biostable artificial analogues (xNTPs) have been reported.^16–25^ This has historically proven challenging due to the high substrate specificity of DNA and RNA polymerases, which – in most cases – must be evolved or engineered^26–28^ to permit DNA-templated XNA polymerisation at full substitution for library synthesis. Although elegant DNA-tagging approaches have been devised to avoid the requirement for an XNA reverse transcriptase (RT),^21,29,30^ where available it may be beneficial to utilise engineered RTs^31^ for XNA-templated cDNA synthesis, enabling efficient amplification and preparation of templates for subsequent rounds of selection.

**Fig. 1:**
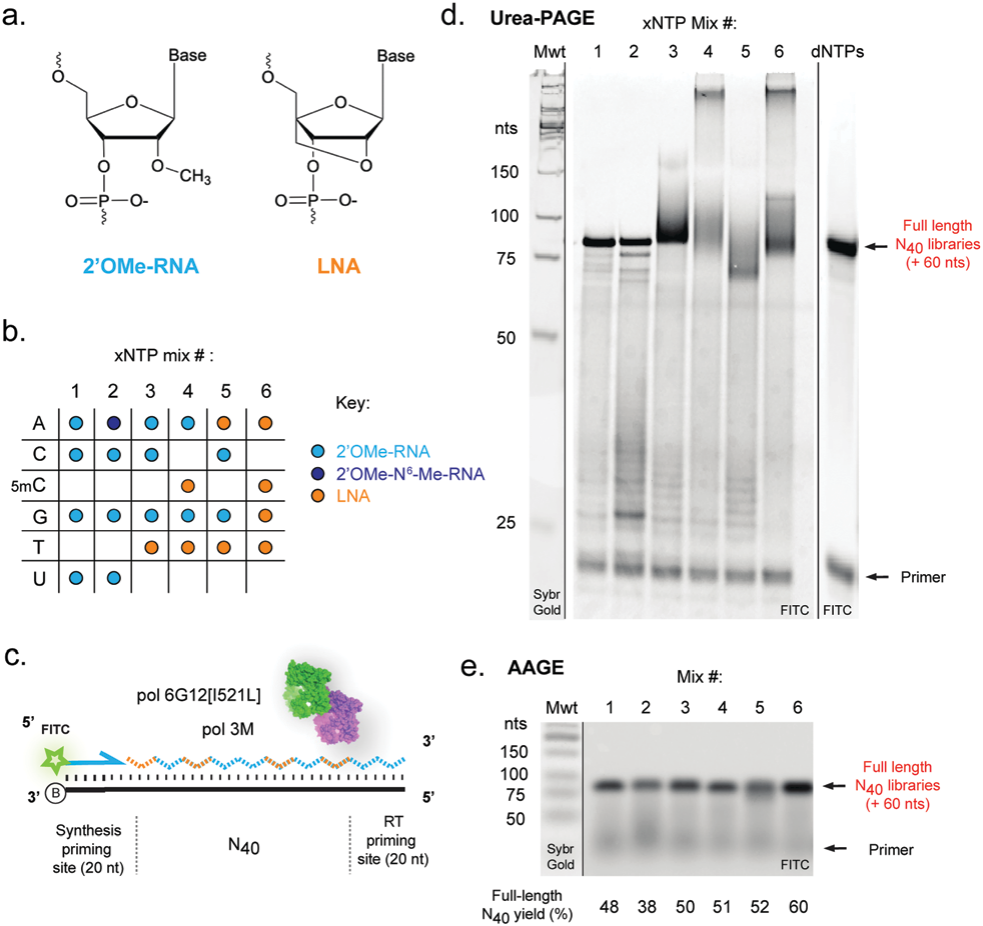
Efficient templated synthesis of pure and mixed-chemistry diverse-sequence 2′OMe-RNA and LNA oligonucleotides. (a) Chemical structures and (b) nucleoside triphosphate (xNTP) combinations of 2′OMe-RNA and LNA used in the study. (c) Diagram and (d) denaturing acrylamide (PAGE) and (e) agarose (AAGE) gels showing DNA-templated synthesis of N_40_ XNA libraries using the chemistries shown in (b), catalysed by a blend of engineered XNA polymerases “pol3M” and “pol6G12[I521L]”. Primer extensions were visualised by FITC fluorescence; unlabelled molecular weight standards (Mwt) were run on the same gels and revealed by staining with Sybr Gold. Gels are representative of at least three replicate reactions per chemistry.

Among the different nucleic acid analogues, 2′-*O*-methyl-RNA (2′OMe-RNA), a natural post-transcriptional modification found in ribosomal, tRNA and mRNA, and 2′-*O*,4′-*C*-methylene-β-d-ribo- or ‘locked’ nucleic acid (LNA)^32,33^ (Fig. 1a) are of particular interest. Both are resistant to serum nucleases and exhibit enhanced binding to complementary RNA and DNA, as well as formation of highly stable secondary structures, due to their conformationally restricted ribose ring structures (resulting from the 2′ methoxy in 2′OMe-RNA and the bridging methylene group in LNA, positioned in the minor groove^34,35^), which reduce the entropic penalty incurred by basepairing. Both 2′OMe-RNA and LNA have been shown to improve target strand invasion and specificity of antisense oligonucleotides, siRNAs and CRISPR/Cas systems, as well as boosting activity of DNAzymes and aptamers in physiological conditions, and are generally well tolerated *in vivo*.^11,36^ Furthermore, 2′OMe-RNA and LNA phosphoramidites are commercially available, enabling scalable chemical synthesis once functional sequences have been identified and optimised. Systems enabling the selection of biostable functional oligonucleotides using such chemistries therefore continue to be key technological goals for the field.

An early example of selections for aptamers composed of 2′OMe-RNA^37^ where libraries were synthesised using a mutant T7 RNA polymerase (T7: Y639F, H784A)^38^ required inclusion of unmodified GTP as well as ‘forcing’ conditions (high xNTP concentrations and Mn^2+^) in order to achieve synthesis of N_30_ libraries. Furthermore, selection seemingly required this chimeric library to be supplemented with chemically-synthesised 2′OMe-RNA.^37^ Reverse transcription of 2′OMe-RNA was possible with Thermoscript (an MMLV RT variant), although inefficient (∼10% cDNA yields on 2′OMe-RNA templates), and the system had a high overall SELEX cycle error rate (51% of amplified cDNAs carried an error).^37^ Subsequently, improvements in 2′OMe-RNA synthesis and RT were achieved using laboratory-evolved mutants of T7,^39–41^ the Stoffel fragment of Taq DNA polymerase^20^ and KOD polymerase,^25^ although these, too, required Mn^2+^ and extended incubation times. Most recently, engineering a two-residue ‘steric gate’ in the Tgo DNA polymerase in order to reduce predicted clashes with bulky 2′-modified nucleotides yielded an efficient 2′OMe-RNA synthetase, “pol2M” and its variant “pol3M”.^42^ Likewise, an efficient 2′OMe-RNA reverse transcriptase was engineered using Tgo by directed evolution, “RT-C8”.^31^ In the case of LNA, a variety of polymerases have been explored for synthesis and RT,^43^ including engineered variants of the polymerases Tgo (“polC7” and “RT521K”, respectively)^17^ and KOD,^25,44^ although LNA in selections has thus far been limited to primer regions^45–47^ or one LNA nucleotide.^25^

Combinations of different XNAs in fully synthetic genetic systems (as demonstrated in ref. 19, 21, 25 and 48) offer a means to navigate a greater variety of chemical and structural space, potentially enabling discovery and evolution of more diverse functional 3D motifs and properties made possible by interactions between alternative polymer chemistries.^49^ Here, we expand the range of mixed-chemistry synthetic genetic systems using two XNA polymerase blends to efficiently synthesise and reverse transcribe mixtures of LNA and/or 2′OMe-RNA.

Beyond sugar modifications, the addition of a variety of side chains to nucleobases, and the creation of alternative basepairs, has proven to be a successful strategy for the expansion of chemical diversity and function of aptamer and DNAzyme reagents.^50–54^ However, their incorporation into selections has thus far been limited to DNA or RNA backbones. Combining base and sugar modifications^55–57^ could conceivably offer a route to synthetic ligands and catalysts with improved function in biological contexts. We therefore also sought to explore the synthesis and reverse transcription of 2′OMe-RNA libraries also bearing a nucleobase modification, 2′-*O*-methyl-*N*^6^-methyl-A aka *N*^6^,2′-*O*-dimethyladenosine (m^6^A_m_), a natural terminal modification of eukaryotic mRNA involved in the regulation of transcript stability.^58^ m^6^A_m_ provides a methyl side-chain that could contribute to formation of hydrophobic paratopes in aptamers evolved to target proteins of interest by analogy to nature's use of m^6^A RNA modifications as sites for recognition by epitranscriptome regulatory proteins.^59^

Typically, synthesis and reverse transcription of XNAs is assayed by primer extension reactions with short, defined DNA templates and incorporation of a limited number of nucleotide analogues. However, to thoroughly evaluate the suitability of our systems for X-SELEX – in particular for more sophisticated directed evolution experiments such as aptamer selections against challenging targets – we examine synthesis and replication (*via* cDNA) of longer, diverse-sequence (N_40_) XNA libraries and assess yields, library diversity and replication fidelity following a complete X-SELEX cycle.

## Results and discussion

We reasoned that although reactions containing mixtures of 2′OMe-RNA and LNA nucleotides would in principle require polymerase phenotypes capable of synthesis using both chemistries, a polymerase capable of efficiently incorporating one set of xNTPs could not necessarily be assumed to be capable of using the other with equal efficiency. Although it has been possible to identify a set of mutations that enable efficient synthesis of both chemistries in a single polymerase scaffold,^25,42^ there are always trade-offs in efficiency and fidelity to be considered. Here, we have sought to explore a complementary approach to optimise synthesis using blends of XNA polymerases engineered in our labs. Blending polymerases is a well-established strategy for improving amplification of long or otherwise challenging DNA templates or in problematic reaction conditions.^60–62^ Screening different combinations of XNA polymerases, we identified a two-polymerase blend that enabled efficient synthesis of mixtures of 2′OMe-RNA and LNA: pol3M^42^ and another Tgo variant, “pol6G12[I521L]”,^18^ previously developed for efficient synthesis of hexitol nucleic acid (HNA) in the absence of Mn^2+^ (Tgo: V93Q, D141A, E143A, A485L, I521L, V589A, E609K, I610M, K659Q, E664Q, Q665P, R668K, D669Q, K671H, K674R, T676R, A681S, L704P, E730G). We initially screened all single- (each LNA-NTP into 2′OMe-NTP mixes) and double-nucleotide combinations (ESI,† Fig. S1), and identified a set of six xNTP mixes (Fig. 1b) that allowed efficient DNA-templated full-length synthesis of diverse-sequence (N_40_) polymers from 2′OMe-RNA primers (Fig. 1c–e) with good yields (expressed as % of yield with dNTPs (ESI,† Fig. S1)): all-2′OMe (75%); 2′OMe-m^6^A, 2′OMe-C, -G, -U (59%); 2′OMe-A, -C, -G, LNA-T (78%); 2′OMe-A, -G, LNA-5mC, -T (80%); LNA-A, -T, 2′OMe-C, -G (81%) and all-LNA (94%). Of the combinations explored, only an LNA purine/2′OMe-RNA pyrimidine mix was inefficiently synthesised (ESI,† Fig. S1).

As we and others have previously observed,^17^ LNA × DNA template (as well as LNA × LNA inter- and intra-molecular) hybridisation can be only incompletely denatured by 8 M urea (and boiling in formamide loading buffer) during polyacrylamide gel electrophoresis (Urea-PAGE), resulting in low mobility species (Fig. 1d). We therefore also analysed samples by alkaline agarose gel electrophoresis (AAGE) and verified that synthesised libraries resolved into bands that were indeed *bona fide* full-length products (Fig. 1e) (subsequently confirmed by sequencing). As expected, all libraries were found to be highly biostable, with minimal degradation observed even after 5 days at 37 °C in 90% human serum (ESI,† Fig. S2), confirming the protection against enzymatic degradation afforded by 2′OMe-RNA and LNA is retained in the chimeric polymers.

To benchmark the pol3M/pol6G12[I521L] polymerase blend against alternative approaches for templated LNA and 2′OMe-RNA synthesis, we compared activity with the recently-described KOD polymerase variant “KOD DGLNK” specifically engineered for 2′OMe-RNA and LNA synthesis^25^ (ESI,† Fig. S3). The blend consistently produced higher yields of pure and mixed-chemistry LNA and/or 2′OMe-RNA N_40_ libraries than KOD DGLNK, in particular in the absence of Mn^2+^ (ESI,† Fig. S3). We note that it remains possible that this performance advantage is at least partly due to our use of 2′OMe-RNA primers, which may be disfavoured by the KOD DGLNK variant.^25^

**Fig. 2:**
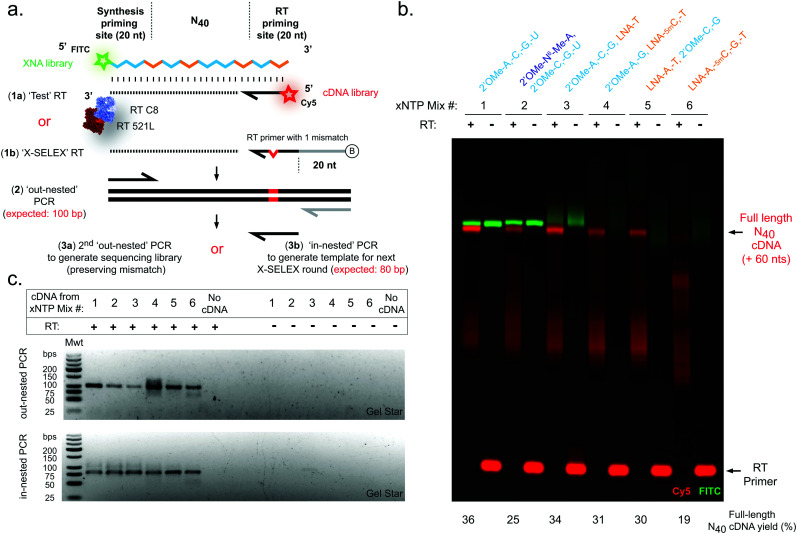
Efficient reverse transcription of pure and mixed-chemistry diverse-sequence 2′OMe-RNA and LNA oligonucleotides. (a) Diagram showing strategies for assessment of XNA RT reactions. (b) Urea-PAGE gel showing XNA-templated (FITC, green) cDNA synthesis (Cy5, red). (c) Agarose gels showing amplification of cDNA by RT-PCR. Gels are representative of at least three replicate reactions per chemistry. RTs were catalysed by a blend of engineered XNA polymerases “RT-C8” and “RT521L” primed either by (a)(1a) a Cy5 fluorophore-labelled DNA primer (‘Test’ RT) in order to directly visualise cDNA synthesis (b), or alternatively RTs were primed by (a)(1b) a biotinylated DNA primer (‘X-SELEX’ RT) enabling purification of cDNA and amplification (c) *via* a two-step PCR strategy: firstly (a)(2) an ‘out-nested’ PCR dependent on a reverse primer site derived from the X-SELEX_RT primer generates templates for, secondly, either (a)(3a) a PCR to generate sequencing libraries preserving a diagnostic mismatched base derived from the RT primer, or instead (a)(3b) an ‘in-nested’ PCR that regenerates the XNA synthesis template (although here is used only as a proof-of-concept demonstration).

Next, we sought to explore reverse transcription of the 2′OMe-RNA/LNA libraries. A screen of XNA reverse transcriptases revealed that again a blend of two previously described RTs, RT-C8^31^ and RT521L^17^ (Tgo: V93Q, D141A, E143A, A385V, E429G, F445L, A485L, I521L, K726R), was capable of efficient synthesis of cDNA templated by N_40_ libraries composed of all six 2′OMe-RNA and/or LNA combinations (Fig. 2), confirmed by both directly imaging cDNA (Fig. 2b) and a two-step semi-nested RT-PCR (Fig. 2a and c), the same amplification strategy used to generate templates for subsequent X-SELEX cycles.^14,15^

In contrast to efficient synthesis, reverse transcription from pure LNA N_40_ templates (Fig. 2b) was clearly more challenging (19% cDNA yield) than pure 2′OMe-RNA (36% cDNA yield). This is not unexpected given that RT-C8 was specifically evolved for 2′OMe-RNA reverse transcription^31^ and neither RT-C8 nor RT521L (identified by screening for activity on templates composed of HNA),^17^ had been optimised for LNA RT. However, the mixed-chemistry libraries prepared using one or two LNA nucleoside triphosphates (with others 2′OMe-NTPs) were well tolerated (34%, 31% and 30% cDNA yield for the [LNA-T], [LNA-5mC, -T] and [LNA-A, -T] mixes, respectively) (Fig. 2b), as was the base-modified 2′OMe-N^6^A-containing mix (25% cDNA yield) (Fig. 2b). Specific amplicons could be easily obtained in PCRs templated by cDNA derived from all RT template chemistries (Fig. 2c). Although these yields suggest that XNA RT is the weakest link in the X-SELEX cycle, all systems nonetheless compare favourably with yields obtained with DNA-templated DNA synthesis assessed using Urea-PAGE (64%; ESI,† Fig. S1), and, as we have explored previously for the full 2′OMe-RNA system,^31^ outperform previously engineered XNA RTs as well as commercially available polymerases.

**Fig. 3:**
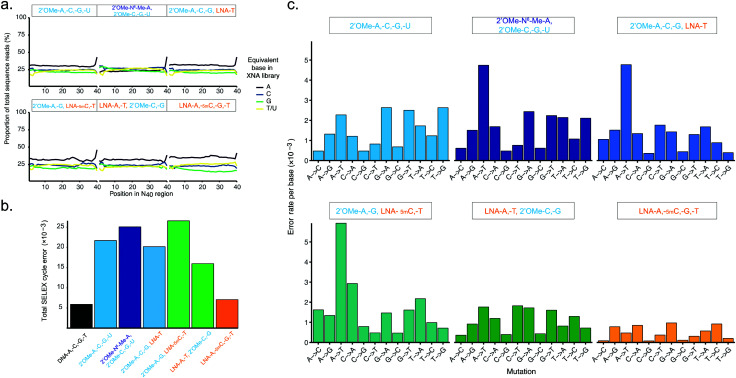
Pure and mixed-chemistry 2′OMe-RNA and LNA synthetic genetic systems enable synthesis and replication of biostable oligonucleotide libraries without substantial biases or loss of library diversity. Graphs show (a) diversity of N_40_ libraries (DNA shown in ESI† Fig. S5), and (b) aggregate error and (c) errors per base following a full cycle of synthesis of pure and mixed-chemistry 2′OMe-RNA and LNA oligonucleotides, reverse transcription and cDNA amplification (DNA → XNA → DNA → DNA sequenced). See also ESI† Tables S2 and S3 for further breakdown of errors and numbers of sequences analysed.

Finally, we sought to confirm whether synthesis and reverse transcription reactions were indeed occurring in a templated manner with sufficient fidelity, and to assess whether sequence diversity (*i.e.* X-SELEX library quality) is maintained by the mixed-chemistry synthetic genetic systems. For these experiments, we chose to examine the complete replication cycle – *i.e.* the sum of synthesis, reverse transcription and cDNA amplification – rather than deconvolute the contributions of the individual polymerases as this aggregate measurement is a closer proxy of a full round of X-SELEX, and the performance of each polymerase has been described elsewhere.^17,18,31,42^ An unbiased defined sequence (“Temp25”) (ESI,† Fig. S4), in addition to N_40_ library sequences (Fig. 1 and 2), were therefore synthesised in each of the six 2′OMe-RNA and/or LNA systems, purified and reversed transcribed, and cDNA amplified to generate barcoded sequencing libraries for multiplexed deep sequencing (Fig. 3). RT reactions were primed using a DNA oligo with a single mismatch design (“XSELEX_RT”; Fig. 2a and ESI,† Table S1, Fig. S4a), which ensured sequences analysed were derived from first-strand cDNA (excluding the possibility of contaminating DNA template from the XNA synthesis step).

Broadly, library diversity derived from the chemically-synthesised DNA template oligonucleotide (ESI,† Fig. S5) was efficiently maintained in all XNA systems (Fig. 3a), indicating a lack of any major biases in synthesis and reverse transcription. In all systems, slight (∼5%) overrepresentation of A was observed (although note that the template DNA N_40_ oligo was found to have slightly higher A content as well (ESI,† Fig. S5)), except when the base-modified 2′OMe-N^6^A was used (Fig. 3a); a spike in As at the 40th nucleotide derives from single nucleotide deletions in either the template oligo (ESI,† Fig. S5) or during XNA synthesis, resulting in the 40th position being the first base of the conserved RT priming site (ESI,† Fig. S4). Conversely, in the pure LNA system, slight (5–10%) underrepresentation of G was observed. It is unclear if these variances are the result of differences in nucleotide analogue incorporation efficiency during synthesis, or are due to errors during reverse transcription, although the observation that nucleotide mixes in which 2′OMe-A was substituted for LNA-A, and/or 2′OMe-G for LNA-G were found to show generally lower synthesis yields than when 2′OMe-A and/or 2′OMe-G were used (ESI,† Fig. S1) would suggest that synthesis is the less efficient step, at least for LNA-G. If so, this may be compensated for by adjustment of the relative nucleotide analogue concentrations, although we did not explore this.

The fidelity of a complete cycle of synthesis and replication using the pol3M/pol6G12[I521L] and RT-C8/RT521L blends (and a blend of Taq and the proof-reading polymerase Deep Vent for DNA amplification) was found to be similar between the pure 2-OMe-RNA (21.7 × 10^−3^) and the mixed-chemistry systems (16.1–26.6 × 10^−3^) (Fig. 3b and ESI,† Table S2), and generally comparable to (total X-SELEX cycle) error rates reported for analogous systems using KOD variants DGLNK and DLK^25^ (15.6 × 10^−3^ for pure 2′OMe-RNA, 28.2 × 10^−3^ for a mixed [2-OMe-A, -C, -G, LNA-T] system).

Interestingly, the mix containing both LNA-A and LNA-T, and the pure LNA system were found to have the lowest total error (16.1 × 10^−3^ and 7.05 × 10^−3^, respectively) (Fig. 3b and ESI,† Table S2). As this seemed remarkably low (comparable to a Tgo DNA-only system^17^ (5.74 × 10^−3^), and lower than the analogous KOD variant system^25^ (14.0 × 10^−3^)), we also verified the fidelity of the pure LNA system by cloning and Sanger sequencing amplicons from the first step ‘out-nest’ PCR (rather than generating Illumina sequencing libraries) and obtained a similar total error (∼7.5 × 10^−3^) (ESI,† Fig. S6).

The error profiles per base (Fig. 3c) suggest that, in the mixed-chemistry systems, misincorporation of As or Ts, and in particular A → T transversions, appear to be the dominant source of error. This is somewhat puzzling given the apparent overrepresentation of As observed in the N_40_ sequences (Fig. 3a) and the apparent efficiency of synthesis of mixes containing 2′OMe-A (ESI,† Fig. S1). However, a possible explanation could be the prevalence of AA dinucleotides (6 instances) and one AAA trinucleotide in the defined Temp25 sequence used for error analysis, which may be more challenging for 2′OMe-RNA systems than single incorporations. As these mixes also contain LNA-T, which appears to be easily incorporated (enabling the highest yields in single-LNA-nucleotide 2′OMe-RNA mixes (ESI,† Fig. S1)), presumably pausing at the di- and trinucleotide positions then raises the incidence of LNA-T × dT mismatches (which appears not to be the case if 2′OMe-U is used instead, except in the system using 2′OMe-N^6^-Me-A, which is presumably more challenging than 2′OMe-A). Indeed, we find that the highest incidences of errors occur at AA(A) positions in the 2′OMe-RNA/LNA mixes (ESI,† Table S3). Replacement of 2′OMe-A with LNA-A, as in the [LNA-A,-T, 2′OMe-C-G] mix, obviates this source of error and produces the highest fidelity mixed-chemistry system (Fig. 3b, c and ESI,† Tables S2, S3).

In the pure LNA system, by contrast, A and T misincorporations are not a major source of errors (Fig. 3c). Likewise, we find no evidence of common (A → G/C → T) errors, which occur with the KOD variant LNA system presumably due to the increased stability of LNA x DNA wobble pairs (dT × LNA-G and/or dG × LNA-T)^25^ (although we cannot rule out that these may be responsible for reduced efficiency of LNA-G incorporation during synthesis, resulting in the underrepresentation of G observed in the all-LNA library (Fig. 3a)). However, a commonly overlooked caveat with such error analyses is the exclusion of early-terminated cDNA. Stalling during reverse transcription appears to occur more frequently with pure LNA templates than the mixed-chemistry or 2′OMe-RNA templates (Fig. 2b and ESI,† Fig. S4c), resulting in cDNA that lacks the forward priming site and therefore fails to be amplified, sequenced and included in the analysis. It cannot therefore be ruled out that such errors do occur in our system, but do not propagate through the replication cycle. The error rates as measured nonetheless bode well for allowing enrichment of functional sequences in X-SELEX experiments, provided selection steps yield sufficient XNA templates to overcome the reduced reverse transcription efficiency; indeed, as we report elsewhere, functional biostable oligonucleotides (‘2′OMezyme’ catalysts) could be readily evolved from an all-2′OMe-RNA system (using pol3M or a functionally similar variant pol2M for synthesis, and RT-C8 for reverse transcription).^42^

## Conclusions

Using blends of previously engineered XNA polymerases, we have established a series of synthetic genetic systems based on mixtures of 2′OMe-RNA and LNA. We report the efficient synthesis and (indirect) replication of diverse-sequence oligonucleotide analogue libraries without major biases in incorporation or reverse transcription. All systems show similar efficiency and fidelity as an all-2′OMe-RNA system, which we have already successfully used to select 2′OMezymes,^42^ and our data suggest that systems in which 2′OMe-NTPs are substituted by one (LNA-T), two (LNA-A,-T) or all four LNA-NTPs offer further improvements. We provide a key proof-of-concept of a system bearing both sugar and nucleobase modifications, suggesting that selections for biostable functional oligos with expanded base chemistry are feasible using commercially available nucleotides (2′OMe-*N*^6^-methyl-ATP, 2′OMe-CTP, -GTP and -UTP), paving the way for development of XNA selection systems with more elaborate side chains.

The establishment of a variety of XNA systems based on highly biostable chemistries offers promising platform technologies for the development of a wide range of biostable functional oligonucleotides for diagnostic and therapeutic applications *in vivo.*^63,64^ The use of oligo libraries fully resistant to serum nucleases minimises or abolishes the need to apply extensive post-selection modifications to prepare resulting functional oligonucleotides for *in vivo* applications (which can negatively affect activity), and enhances the prospects for selections in more realistic biological settings: in live cell or organoid culture or even in whole animals.^65^ Excitingly, such approaches may offer the prospect of selections for aptamers capable of cell- or organ-specific delivery, and could enable direct screening for modulators of biological phenotype, a crucial approach in the immunoglobulin antibody discovery pipeline,^66^ but which has yet to be implemented for oligonucleotide aptamers and catalysts.

## Author contributions

A. I. T. conceived and directed the project. J. R. D. H. acquired, curated and analysed data with A. I. T. and G. D. Polymerases were supplied by N. F., G. H. and P. H. A. I. T. and J. R. D. H. wrote the manuscript with contributions from the other authors.

## Conflicts of interest

There are no conflicts to declare.

## Supplementary Material

CB-003-D2CB00035K-s001
