# Supplementary material for: Efficient synthesis and replication of diverse sequence libraries composed of biostable nucleic acid analogues
Source: RSC Chem Biol. 2022 Aug 30;3(10):1209–15. doi: 10.1039/d2cb00035k (PMC9533476; doi:10.1039/d2cb00035k)
Supplement: CB-003-D2CB00035K-s001 [file CB-003-D2CB00035K-s001.pdf]

Electronic Supplementary Material (ESI) for RSC Chemical Biology.
This journal is © The Royal Society of Chemistry 2022

Supplementary Material for:

Efficient synthesis and replication of diverse sequence libraries composed of biostable nucleic acid analogues

John R. D. Hervey [a], Niklas Freund [b], Gillian Houlihan [b], Gurpreet Dhaliwal [a], Philipp Holliger *[b] and Alexander I. Taylor * [a]

[a] Cambridge Institute of Therapeutic Immunology & Infectious Disease (CITIID), University of Cambridge, Cambridge CB2 0AW, United Kingdom

[b] Medical Research Council Laboratory of Molecular Biology, Cambridge CB2 0QH, United Kingdom

*corresponding authors:

ait29@cam.ac.uk

ph1@mrc-lmb.cam.ac.uk

Contents:

Supplementary Figures 1 – 6.

Supplementary Tables 1 & 2.

Materials and Methods.

Supplementary References.

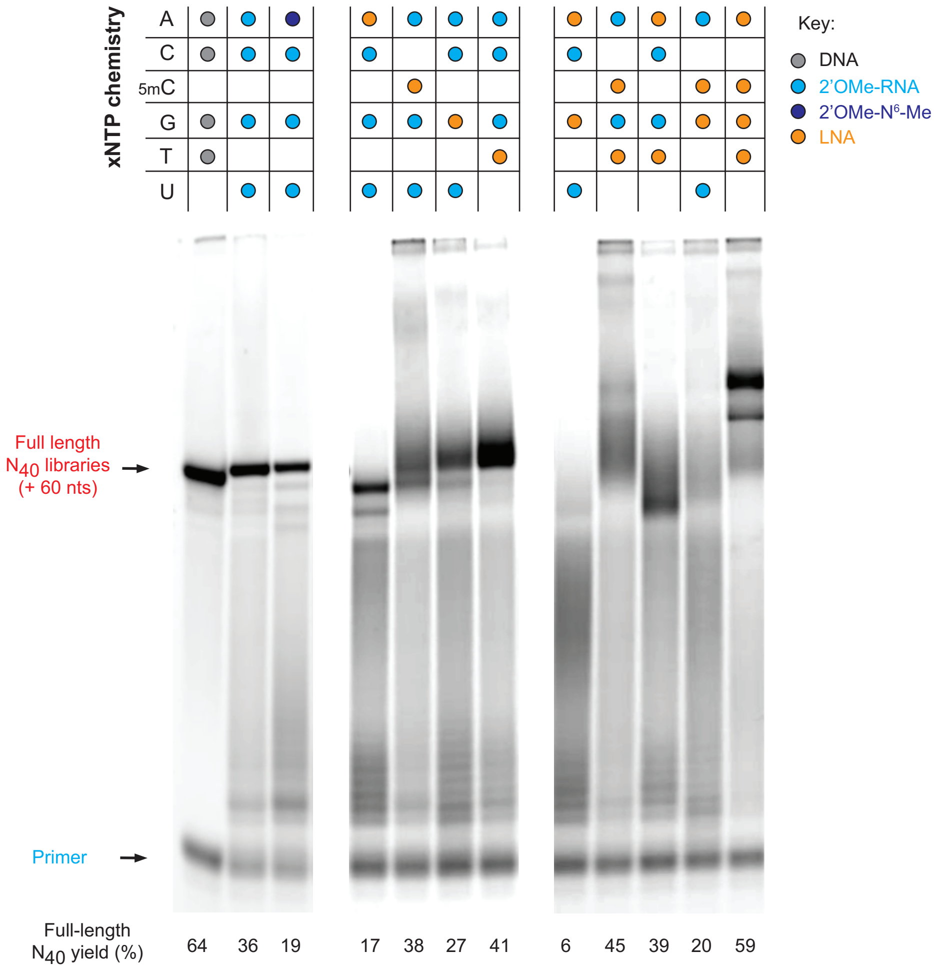

Supplementary Figure 1. Synthesis of N_{40} X-SELEX libraries using mixtures of 2'OMe-RNA and LNA nucleotides.

Urea-PAGE gel showing synthesis of random-sequence (N_{40}) libraries using DNA template "N40_temp, 2'OMe-RNA primer "2OMe_Fw" (See Supplementary Table 1) and a blend of polymerases "pol3M"[1] and "pol6G12[I521L]"[2], using mixtures of 2'OMe-RNA and/or LNA nucleotide triphosphate analogues (xNTPs) as shown.

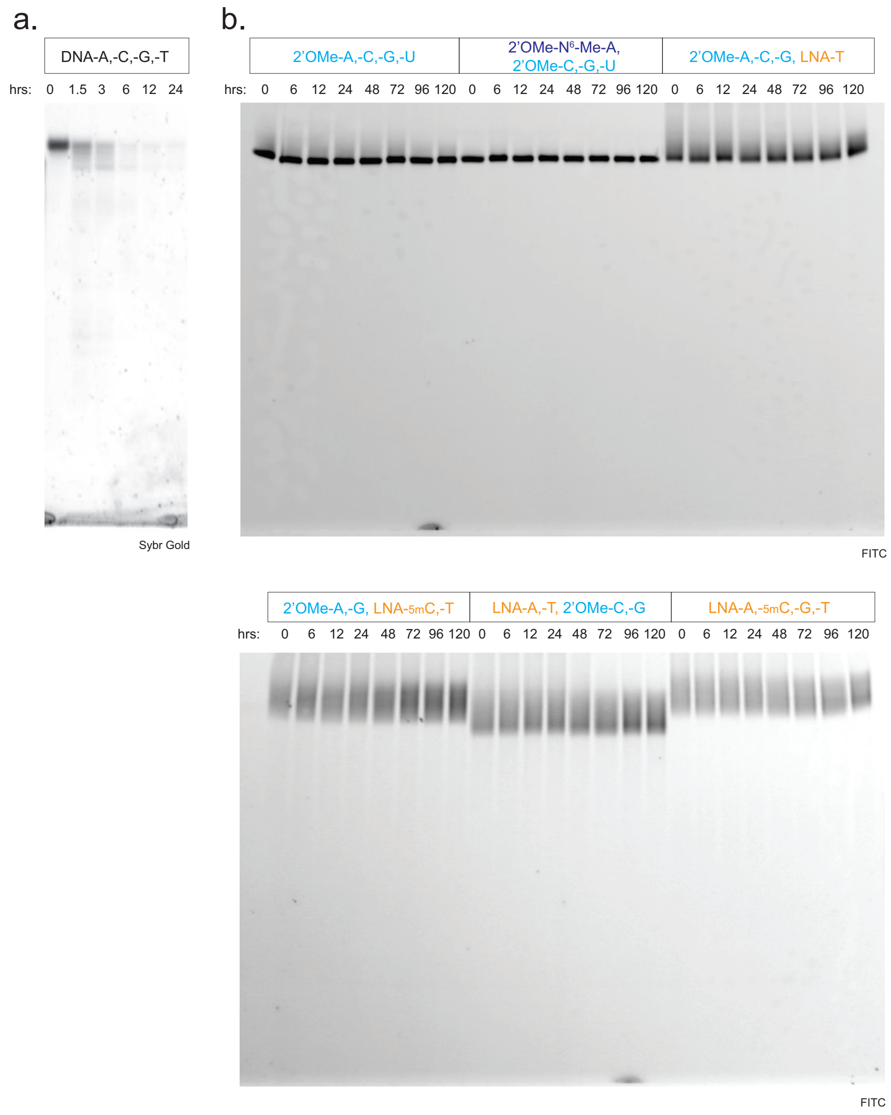

Supplementary Figure 2. 2'OMe-RNA, LNA and mixed-chemistry libraries are fully resistant to serum nucleases.

*Urea-PAGE gels showing timecourses of (**A**) DNA control and (**B**) 2'OMe-RNA and/or LNA N_{40} libraries incubated at 37 °C in 90% human serum.*

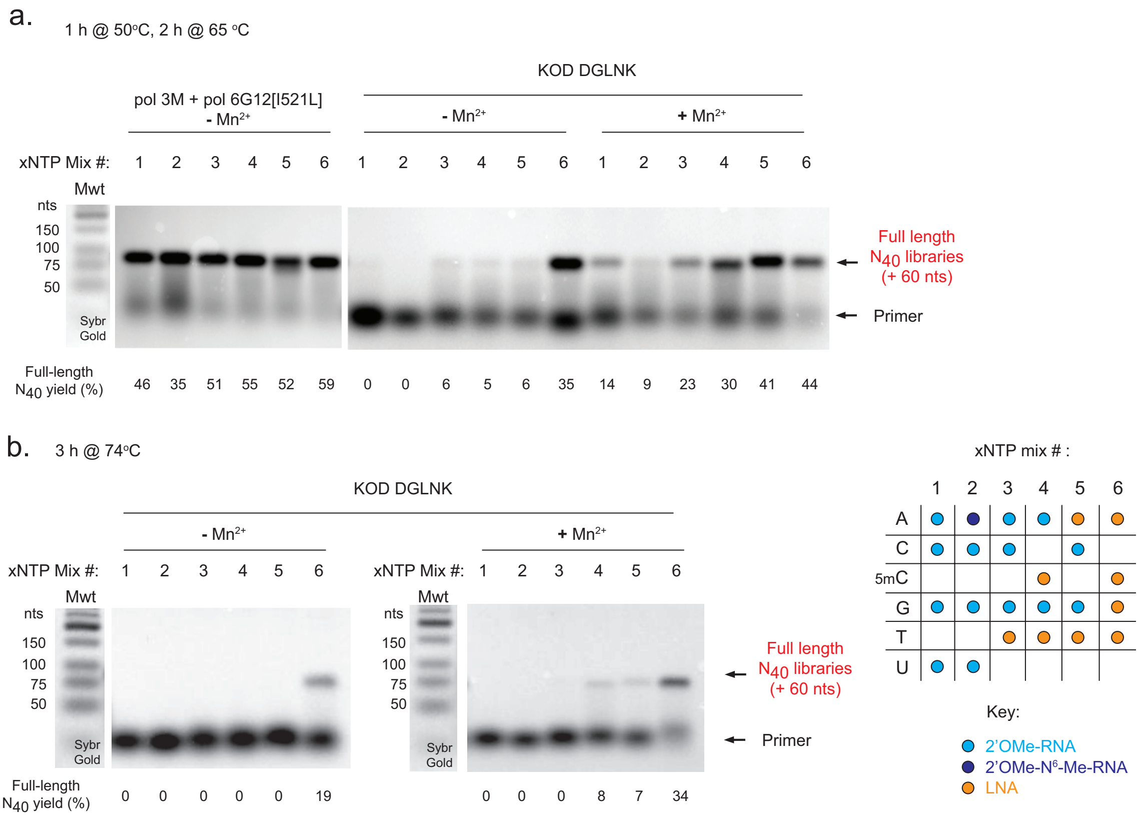

Supplementary Figure 3. Comparison of 2'OMe and LNA N_{40} SELEX library synthesis using an alternative polymerase.

*(**A** and **B**) Alkaline agarose (AAGE) gels showing 2'OMe-RNA, LNA and mixed 2'OMe/LNA random-sequence (N_{40}) library synthesis using DNA template "N40_temp" and 2'OMe-RNA primer "2OMe_Fw" (See Supplementary Table 1), and either a blend of polymerases "pol3M"[1] and "pol6G12[I521L]"[2], or polymerase "KOD DGLNK"[3] with or without 1 mM $MnSO_4$ (-/+ Mn^{2+}), which is required for KOD DGLNK-mediated synthesis of 2'OMe-RNA[3]. Reactions were performed using (**A**) synthesis temperatures previously reported for Tgo polymerase mutants[2, 4] or (**B**) KOD polymerase mutants[3] as shown. The blend of polymerases pol3M and pol6G12[I521L] produces higher yields of full-length libraries with all mixtures of 2'OMe-RNA and LNA xNTPs shown compared with polymerase KOD DGLNK, and does not require Mn^{2+}* for *efficient synthesis.*

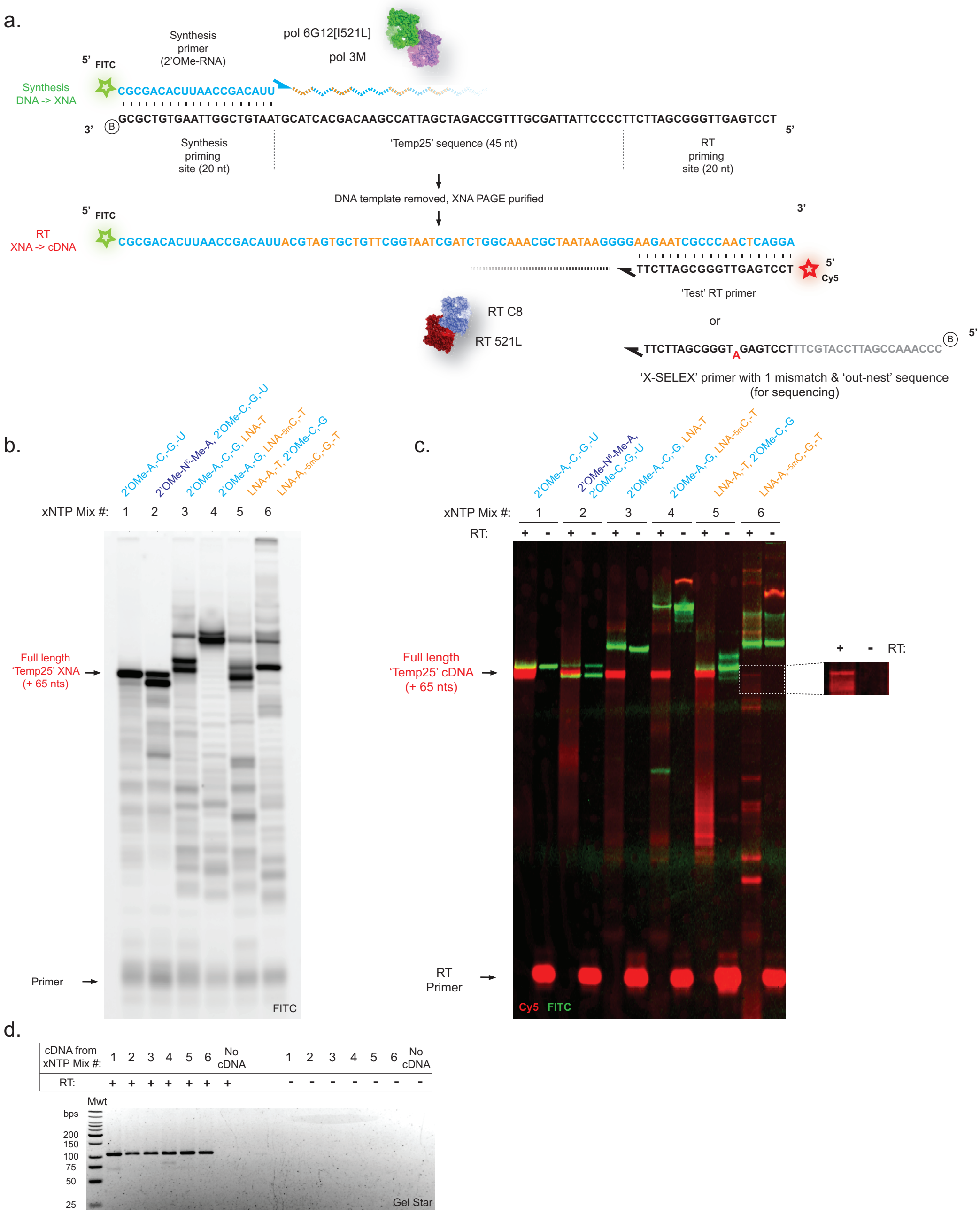

Supplementary Figure 4. Synthesis and reverse transcription of defined 2'OMe-RNA, LNA and mixed-chemsitry sequences for determination of replication fidelity.

*(**A**) Scheme for synthesis and reverse transcription (RT) of defined-sequence XNAs composed of 2'OMe-RNA, LNA or 2'OMe-RNA/LNA mixtures. Synthesis of XNA polymers containing the 'Temp25' sequence was performed using DNA template "Temp25_temp" (shown in black) and (FITC-labelled) 2'OMe-RNA primer "2OMe_Fw" (shown in cyan) (See Supplementary Table 1), and verified by (**B**) Urea-PAGE. Following removal of the (biotinylated) DNA template by streptavidin bead capture and treatment with DNase I, full-length sequences were PAGE purified and reverse transcribed using a blend of XNA-dependent DNA polymerases "polC8"[5] and "polI521L"[4]. To verify synthesis of first-strand cDNA, either (Cy5-labelled) "Test_RT" primer (See Supplementary Table 1) was used during RT and cDNA directly imaged by (**C**) Urea-PAGE, or a longer primer, "XSELEX_RT" (See Supplementary Table 1), was used, containing an 'out-nest' sequence absent from the original DNA template, enabling specific amplification by (**D**) semi-nested PCR. The amplicons in this PCR provided the templates for generation of libraries for deep sequencing. A mismatch in the XSELEX_RT primer enables verification that cDNA sequences derive from XNA RT and not amplification of the original DNA template. Fidelity results are shown in Figure 5 and Supplementary Table 2.*

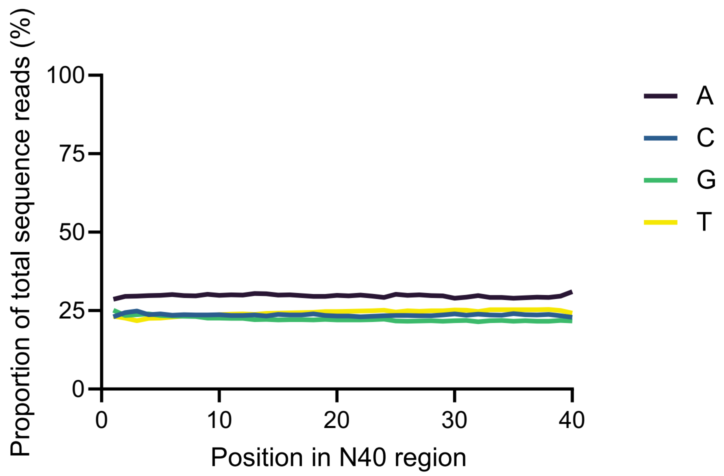

Supplementary Figure 5. Diversity of template used for XNA library synthesis.

Diversity of N_{40} region in DNA template oligonucleotide used for XNA library synthesis ("N40_temp") determined by deep sequencing.

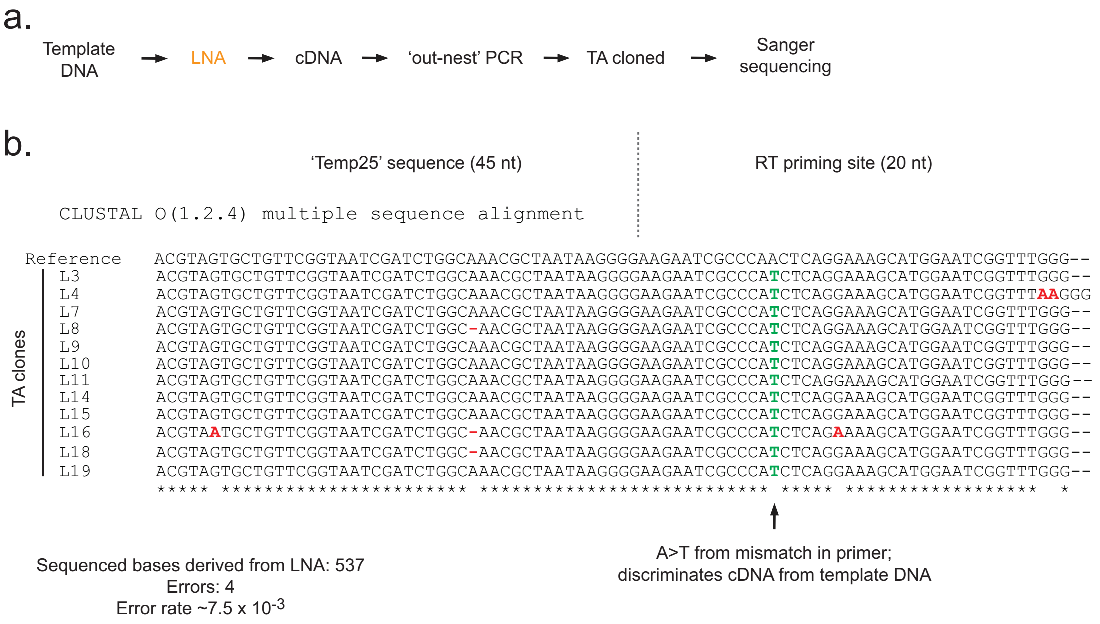

Supplementary Figure 6. Sanger sequencing of cDNA reverse transcribed from all-LNA sequences.

*(**A**) All-LNA polymers containing the 'Temp25' sequence were synthesised using DNA template "Temp25_temp" (See Supplementary Table 1), then reverse transcribed using primer "XSELEX_RT" (See Supplementary Table 1). cDNA was amplified and cloned into plasmids in E.coli, and colonies cultured to prepare plasmid DNA. (**B**) Sanger sequences of clones yielding sufficient plasmid and high-quality reads. The occurrence of errors in the 'Temp25' region (derived from LNA) are consistent with the total error rate for the all-LNA system determined by deep sequencing (Figure 5 and Supplementary Table 2) (7.05×10^{-3}).*

Name	Sequence (5′ → 3′)	Modifications	Notes
N40_temp	TCCTGAGTTGGGCGATTCTTNNNNNNNNNNNNNNNNNNNNNNNNNNNNNNNNNNNNNNNNAATGTCGGTTAAGTGTCGCG	3′ Biotin-TEG	Template for synthesis of degenerate (N40) XNA libraries.
Temp25_temp	TCCTGAGTTGGGCGATTCTTCCCCTTATTAGCGTTTGCCAGATCGATTACCGAACAGCACTACGTAATGTCGGTTAAGTGTCGCG	3′ Biotin-TEG	Template for synthesis of defined ‘Temp25’ XNA sequence for error analysis.
2OMe_Fw	CGCGACACUUAACCGACAUU	5′ 6FAM	Forward primer used for XNA synthesis.
Test_RT	TCCTGAGTTGGGCGATTCTT	5′ Cy5	Primer used for reverse transcription when analysed by Urea-PAGE.
XSELEX_RT	CCCAAACCGATTCCATGCTTTCCTGAGATGGGCGATTCTT	5′ Biotin-TEG	Primer used for reverse transcription for preparation of sequencing libraries. Red base indicates mismatch to synthesis templates, for identifying reverse transcribed reads.
Fwd	CGCGACACTTAACCGACATT	none	Forward primer used for out-nest and in-nest amplification.
OUTnest_Rev	CCCAAACCGATTCCATGCTT	none	Reverse primer used for out-nest amplification.
INnest_Rev	TCCTGAGTTGGGCGATTCTT	none	Reverse primer used for in-nest amplification.
i5_Bar1_Fw	AATGATACGGCGACCACCGAGATCTACACTCTTTCCCTACACGACGCTCTTCCGATCTNNNATCACGCGCGACACTTAACCGACATT	none	Forward primer for making sequencing libraries with Illumina adapters, with barcode 1 (underlined).
i5_Bar2_Fw	AATGATACGGCGACCACCGAGATCTACACTCTTTCCCTACACGACGCTCTTCCGATCTNNNCGATGTCGCGACACTTAACCGACATT	none	Forward primer for making sequencing libraries with Illumina adapters, with barcode 2 (underlined).
i5_Bar3_Fw	AATGATACGGCGACCACCGAGATCTACACTCTTTCCCTACACGACGCTCTTCCGATCTNNNTTAGGCCGCGACACTTAACCGACATT	none	Forward primer for making sequencing libraries with Illumina adapters, with barcode 3 (underlined).
i5_Bar4_Fw	AATGATACGGCGACCACCGAGATCTACACTCTTTCCCTACACGACGCTCTTCCGATCTNNNTGACCACGCGACACTTAACCGACATT	none	Forward primer for making sequencing libraries with Illumina adapters, with barcode 4 (underlined).
i5_Bar5_Fw	AATGATACGGCGACCACCGAGATCTACACTCTTTCCCTACACGACGCTCTTCCGATCTNNNACAGTGCGCGACACTTAACCGACATT	none	Forward primer for making sequencing libraries with Illumina adapters, with barcode 5 (underlined).
i5_Bar6_Fw	AATGATACGGCGACCACCGAGATCTACACTCTTTCCCTACACGACGCTCTTCCGATCTNNNGCCAATCGCGACACTTAACCGACATT	none	Forward primer for making sequencing libraries with Illumina adapters, with barcode 6 (underlined).

i5_Bar7_Fw	AATGATACGGCGACCACCGAGATCTACACTCTTTCCCTACACGACGCTCTTCCGATCTNNNCAGATCCGCGACACTTAACCGACATT	none	Forward primer for making sequencing libraries with Illumina adapters, with barcode 7 (underlined).
i7_Rev	CAAGCAGAAGACGGCATACGAGATGTGACTGGAGTTCAGACGTGTGCTCTTCCGATCCCCAAACCGATTCCATGCTT	none	Reverse primer for making sequencing libraries with Illumina adapters.

Supplementary Table 1. Oligonucleotide sequences used in the study. *Black indicates DNA oligo, cyan indicates 2’OMe-RNA oligo.*

Chemistry	Bases Sequenced	Total Error (x 10^{-3})
All DNA	Defined seq: 1.24 kb (from ref [4])	5.74
	N_{40}: 1,061,335	-
All 2'OMe-RNA	Defined seq: 885,370	21.7
	N_{40}: 4,530,560	-
2'OMe-N^6-mA, 2'OMe-C, 2'OMe-G, 2'OMe-U	Defined seq: 476,578	25.2
	N_{40}: 3,367,200	-
2'OMe-A, 2'OMe-C, 2'OMe-G, LNA-T	Defined seq: 771,621	20.3
	N_{40}: 3,626,640	-
2'OMe-A, LNA-$_{5m}$C, 2'OMe-G, LNA-T	Defined seq: 657,440	26.6
	N_{40}: 2,140,880	-
LNA-A, 2'OMe-C, 2'OMe-G, LNA-T	Defined seq: 297,263	16.1
	N_{40}: 2,218,360	-
All LNA	Defined seq: 1,367,267	7.05
	N_{40}: 2,319,160	-

Supplementary Table 2. Deep sequencing reads used to determine XNA library diversity and aggregate error per X-SELEX cycle (total error).

		Chemistry					
Position (Primer+)	Reference sequence	2'OMe-A,-C,-G,-U	2'OMe-N^6-Me-A, 2'OMe-C,-G,-U	2'OMe-A,-C,-G, LNA-T	2'OMe-A,-G, LNA-5mC,-T	LNA-A,-T, 2'OMe-C,-G	LNA-A,- 5mC,-G,-T
1	A	Below sequencing error	Below sequencing error	Below sequencing error	Below sequencing error	Below sequencing error	Below sequencing error
2	C						
3	G						
4	T						
5	A						
6	G	1.3E-02	1.2E-02	4.7E-03	8.1E-03	1.0E-02	3.2E-03
7	T	2.1E-02	2.0E-02	3.0E-03	2.9E-03	4.4E-03	1.3E-02
8	G	9.0E-03	9.6E-03	8.5E-03	6.9E-03	9.3E-03	8.8E-03
9	C	9.1E-03	8.8E-03	6.7E-03	1.7E-03	8.5E-03	2.8E-03
10	T	3.2E-02	3.0E-02	5.8E-03	6.2E-03	7.0E-03	7.1E-03
11	G	3.0E-02	2.9E-02	1.6E-02	1.8E-02	2.1E-02	4.6E-03
12	T	2.9E-02	2.8E-02	5.2E-03	8.5E-03	4.9E-03	6.1E-03
13	T	2.0E-02	1.8E-02	4.6E-03	4.8E-03	5.0E-03	7.2E-03
14	C	7.5E-03	7.0E-03	2.4E-02	4.8E-03	2.4E-02	5.9E-03
15	G	1.7E-02	1.5E-02	1.1E-02	2.6E-02	1.4E-02	5.7E-03
16	G	5.2E-02	4.9E-02	1.8E-02	2.3E-02	2.0E-02	5.0E-03
17	T	3.1E-02	2.4E-02	8.3E-03	6.1E-03	7.3E-03	4.1E-03
18	A	1.4E-02	2.8E-02	9.5E-02	9.7E-02	2.3E-02	6.2E-03
19	A	1.7E-02	3.2E-02	5.3E-02	1.3E-01	3.3E-02	5.8E-03
20	T	1.4E-02	1.3E-02	5.8E-03	4.8E-03	3.9E-03	5.1E-03
21	C	1.2E-02	1.1E-02	3.3E-02	7.3E-03	1.9E-02	4.3E-03
22	G	2.9E-02	3.8E-02	1.5E-02	4.8E-02	2.5E-02	3.0E-03
23	A	2.6E-02	6.1E-02	1.9E-02	2.1E-02	9.4E-03	5.6E-03
24	T	2.0E-02	1.2E-02	8.9E-03	2.8E-02	1.5E-02	6.1E-03
25	C	1.2E-02	1.1E-02	2.4E-02	3.5E-03	3.1E-02	3.7E-03
26	T	2.7E-02	2.2E-02	8.9E-03	9.0E-03	9.9E-03	1.1E-02
27	G	7.6E-03	8.2E-03	1.7E-02	1.6E-02	2.2E-02	6.4E-03
28	G	1.8E-02	1.7E-02	2.0E-02	1.3E-02	1.1E-02	1.1E-02
29	C	7.1E-03	8.4E-03	6.6E-03	3.4E-03	8.6E-03	3.4E-03
30	A	4.1E-02	7.5E-02	5.0E-02	1.4E-01	3.9E-02	3.9E-02
31	A	1.7E-02	2.8E-02	1.7E-02	4.8E-02	1.4E-02	7.4E-03
32	A	1.2E-02	1.7E-02	1.0E-02	6.0E-03	5.2E-03	4.2E-03
33	C	5.0E-03	4.1E-03	4.0E-03	2.2E-03	1.5E-02	3.5E-03
34	G	1.6E-02	1.1E-02	9.6E-03	8.2E-03	9.6E-03	6.6E-03
35	C	1.3E-02	9.5E-03	7.6E-03	4.7E-03	5.7E-03	1.7E-03
36	T	3.6E-02	2.4E-02	7.9E-03	5.0E-03	9.6E-03	3.3E-03
37	A	2.9E-02	4.9E-02	7.0E-02	4.9E-02	1.6E-02	7.4E-03
38	A	1.5E-02	2.4E-02	2.1E-02	1.6E-02	6.8E-03	3.9E-03
39	T	1.9E-02	1.4E-02	3.3E-03	4.1E-03	7.0E-03	3.1E-03
40	A	1.5E-02	2.5E-02	4.4E-02	5.2E-02	1.9E-02	6.7E-03
41	A	2.0E-02	2.9E-02	2.5E-02	2.4E-02	1.2E-02	5.6E-03
42	G	5.8E-03	2.9E-03	2.4E-03	1.6E-03	7.0E-03	1.8E-03
43	G	5.1E-03	3.1E-03	3.3E-03	2.3E-03	3.0E-03	1.5E-03
44	G	1.2E-02	9.8E-03	1.2E-02	1.1E-02	1.1E-02	1.6E-03
45	G	1.6E-02	1.1E-02	1.1E-02	9.9E-03	1.9E-02	3.2E-03

Supplementary Table 3. Aggregate error per X-SELEX cycle (total error) by position.

Materials and methods

All chemicals were supplied by Merck / MilliporeSigma (Germany) unless stated otherwise.

Nucleotides and oligonucleotides. Triphosphates of LNA (LNA-NTPs; LNA-ATP, LNA-5mCTP, LNA-GTP, LNA-TTP) were kindly provided by Prof. J. Wengel (University of Southern Denmark), triphosphates of 2'OMe-RNA (2'OMe-NTPs; 2'OMe-ATP, 2'OMe-CTP, 2'OMe-GTP, 2'OMe-UTP) were obtained from Jena Biosciences (Germany), 2'OMe-N^6-methyl-A triphosphate was obtained from Trilink Biotechnologies (USA) and triphosphates of DNA (Illustra dNTPs) from GE Life Sciences (USA). Oligonucleotides were synthesised by Integrated DNA Technologies (Belgium) or Merck / MilliporeSigma (Germany).

Synthesis of XNA from DNA template. XNA library synthesis and purification was performed as described in [6, 7]; in brief: A reaction mixture consisting of 1 × ThermoPol buffer (New England BioLabs, cat. no. B9004), 2 μM 3′ biotinylated DNA template ("N40_temp", or "Temp25_temp" Supplementary Table 1), 1 μM 2'OMe-RNA primer ("2OMe_Fw" Supplementary Table 1), 125 μM each of LNA-NTPs or 2'OMe-NTPs, or equimolar mixtures, and a polymerase blend containing 1 μM each of the XNA polymerases "pol3M"[1] and pol6G12[I521L][2] was incubated 1 h at 50 °C followed by 2 h at 65 °C. Reaction mixes were heated to 95 °C for 2 minutes and then immediately transferred to ice before addition of enzyme. Typical reaction sizes were 10 μL for gel-based analysis only, or 400 μL for purification, reverse transcription, sequencing etc., unless otherwise indicated. For reaction mixtures containing LNA nucleotides, 4 % (v/v) single-stranded DNA binding protein (ET-SSB, New England BioLabs, cat. no. M2401) was also included in the reaction. For comparisons with polymerase KOD DGLNK[3], reactions were performed in KOD Buffer #2[3] (120 mM Tris-HCl pH 8.8, 10 mM KCl, 6 mM $(NH_4)_2SO_4$, 0.1% Triton X-100, 0.001% BSA, 2.5 mM $MgSO_4$), with or without supplementation with 1 mM $MnSO_4$ (added after the template and primer annealing step), using the same thermal cycling conditions as above, or by incubation for 3 h at 74 °C.

Purification of synthesised XNA. Streptavidin bead capture was used to remove the biotinylated DNA template, followed by DNaseI digestion and urea-PAGE to further purify synthesised XNA, as described previously[6, 7]. Briefly, streptavidin MyOne C1 Dynabeads (Invitrogen/Life Technologies, cat. no. 65001) (2.5 mg beads per 400 μL rxn / 800 pmol target) were washed with streptavidin bead bind and wash buffer (BwB-Tw: 5 mM Tris-HCl pH 7.5, 0.5 mM EDTA, 1 M NaCl, 0.1% v/v Tween 20). XNA synthesis reaction mixtures were mixed with 5 × BwB-Tw to bring the concentration to 1 × and then incubated on the washed beads at 4 °C on a rotary shaker for 16 hours. The beads were washed five times with 1 × BwB-Tw, once with nuclease-free H_2O (Qiagen, cat. no. 129114), and then single stranded XNA was eluted from the bead-template using 0.1 M NaOH and neutralised with 1 M Tris-HCl pH 7.5. TURBO DNase I buffer (10 ×, Invitrogen, cat. no. AM2238) was added to the eluted single-stranded XNA preparations to 1 × with 0.1 U/μL TURBO DNase I, incubated at 37 °C for 2 hours and then inactivated at 80 °C for 20 minutes. Urea-PAGE was performed as described below, and excised gel slices were pulverised using a Scientific Laboratory Supplies VelociRuptor V2 Microtube Homogeniser and 5 mm nuclease-free stainless-steel ball

bearings (Qiagen, cat. no. 69989) for 30 seconds. Nuclease-free H_2O was added to create a pourable slurry (~1000 μL), rapidly freeze-thawed with dry ice and a 99 °C incubator, and then incubated for 16 hours on a rotary mixer at room temperature. The solution was separated from the gel matrix using 0.45 μm spin filters (Spin-X, Corning cat. no. 8163) and then the purified single stranded XNA was finally retrieved by precipitation in ice cold ethanol, washed with 70% ethanol and dried in a vacuum concentrator.

Reverse transcription (RT) of DNA from XNA templates. Reaction mixtures consisting of: 0.1 μM purified XNA, 1 × ThermoPol buffer supplemented with an additional 2 mM $MgSO_4$ (New England BioLabs, cat. no. B1003), 500 μM each dNTP, 0.1 μM RT primer (either "Test_RT" for analysis of cDNA by urea-PAGE, or "XSELEX_RT" for analysis of cDNA by sequencing, Supplementary Table 1), and a polymerase blend containing 0.2 μM each of the XNA-dependent DNA polymerases "polC8"[5] and "polI521L"[4] were incubated at 65 °C for 12 hours. Reaction mixes were heated to 95 °C for 2 minutes and then immediately transferred to ice prior to addition of enzyme. First-strand cDNA products from RT reactions performed with 5′ biotinylated "XSELEX_RT" primer were purified by bead-capture prior to PCR amplification and sequencing library preparation: Streptavidin MyOne C1 Dynabeads were washed three times with 1 × BwB-Tw. RT reaction mixes were diluted 1:5 with nuclease-free H_2O and 5 × BwB-Tw to bring the concentration to 1 × and then incubated on the washed beads at 4 °C on a rotary shaker for 16 hours. The beads were washed three times with 1 × BwB-Tw and then the XNA template was removed by washing once with 0.1 M NaOH. The beads were washed with 1 × BwB-Tw supplemented with 50 mM Tris-HCl pH 7.5 and then again with nuclease-free H_2O. Finally, the single-stranded cDNA was eluted from the beads in H_2O incubated at 80 °C for 10 minutes, and purified by ethanol precipitation, washed with 70% ethanol and dried in a vacuum concentrator.

PCR amplification of cDNA. PCR reactions were performed using OneTaq® Hot Start Quick-Load® 2X Master Mix with Standard Buffer (New England BioLabs, cat. no. M0488) in accordance with the manufacturer's directions. For 'out-nested' PCR, 0.2 μM each primer "Fwd" and "OUTnest_Rev" (Supplementary Table 1) were used; for 'in-nested' PCR, 0.2 μM each primer "Fwd" and "INnest_Rev" (Supplementary Table 1) were used, with cycling conditions 30s at 94 °C, 10-20 cycles of [30s at 94 °C, 30s at 57 °C, 30s at 68 °C], 5 min at 68 °C.

Denaturing poly-acrylamide gel electrophoresis. Urea-PAGE gels were prepared with 8 M urea, 1 × TBE buffer (3), 15 % (w/v) acrylamide (diluted from 40 % w/v acrylamide/bis-acrylamide, 19:1, Severn Biotech, cat. no. 20-2400-10), 0.05 % w/v ammonium persulphate (APS), and 0.2 % v/v N,N,N′,N′-tetramethylethylenediamine (TEMED; Sigma-Aldrich, T7024). Gels were run in 1 × TBE buffer at 10 W for 10 minutes followed by 24 W for 1.5 hours. Gel loading buffer was 95 % (v/v) formamide, 10 mM Tris-HCl pH 7.4, 50 mM EDTA, and 0.01 % (w/v) bromophenol blue, and the samples were heated to 99 °C for 10 minutes prior to loading. The DNA molecular weight marker was Low Molecular Weight ladder, New England Biolabs, cat. no. N3233. Gels were imaged using a Fujifilm FLA-5000 fluorescence gel imager using Cy2 and Cy5 settings, and, where indicated, subsequently stained with SYBR Gold nucleic acid stain (Life Technologies, cat. no. S-11494) and imaged again with Cy2 settings.

Alkaline agarose gel electrophoresis. Alkaline agarose gels were run in a buffer consisting of 50 mM NaOH, and 1 mM EDTA. The gels were prepared at 4 % (w/v) agarose in the same buffer and run at 100 V (3.5 V/cm) for 1.5 hours. Samples were prepared in Purple Loading Dye (New England Biolabs, cat. no. B7025) supplemented with 100 mM NaOH, and heated to 99 °C for 10 minutes prior to loading.

Serum stability assay. 0.5 pmol each N40 XNA library was incubated in 90 % human serum (Sigma-Aldrich / Merck) at 37 °C with samples taken at 0, 6, 12, 24, 48, 72, 96, and 120 hours, mixed with mixed with excess gel loading buffer, snap frozen on dry ice, and stored at -80 °C prior to urea-PAGE analysis.

Preparation of sequencing libraries. Sequencing libraries were constructed by appending Illumina adapter sequences in a second 'out-nested' PCR. 'Out-nest' PCR amplicons (see above) were treated with ExoSAP-IT (Thermo Fisher Scientific, USA) according to the manufacturer's instructions, then used as template in a PCR reaction (with conditions as described above) with primers "i5_Barx_Fw" and "i7_Rev" (Supplementary Table 1). 200 μL PCR reactions were purified using a QIAquick PCR & Gel cleanup kit (Qiagen, cat. no. 28506). Sequencing was performed using either an Illumina HiSeq or MiSeq system, using a Micro Kit v2 (300-cycles) (Illumina, cat. no. MS-103-1002) according to the manufacturer's instructions.

Analysis of sequencing data. Raw paired-end Illumina sequencing reads were joined using PEAR[8] and then processed using the Galaxy server[9], BowTie2[10] and BWA-MEM[11]. Reads were split by chemistry using the Barcode Splitter tool and then trimmed to the N40 or Temp25 region plus the RT primer binding site. Base incorporation percentages were calculated using WebLogo3[12] in probability mode and plotted using R. Temp25 reads were filtered using Cutadapt to select only reads containing a mismatched base derived from the "XSELEX_RT" reverse transcription primer (Supplementary Table 1), in order to ensure reads were derived from cDNA and not template DNA contamination. Error analysis was performed as described previously[13] using code available at: https://github.com/holliger-lab/fidelity-analysis.

Sanger sequencing. Out-nest PCR amplicons were cloned into the pCR™4-TOPO™ Vector using the TOPO™ TA Cloning™ Kit for Sequencing (Invitrogen, cat. no. 450071) according to the manufacturer's instructions. Colonies were picked and inserts verified via colony PCR. Successful transformants were cultured in LB and plasmids extracted using a Monarch® Plasmid Miniprep Kit (New England Biolabs, cat. no. T1010). Plasmids meeting sample specifications were Sanger sequenced by Source BioScience (UK) using standard M13R primers, and resulting Temp25 sequences were aligned to the reference using Clustal Omega[14].

Supplementary References

1. N. Freund, A. I. Taylor, S. Arangundy-Franklin, N. Subramanian, S.-Y. Peak-Chew, M. Abramov, P. Herdewijn and P. Holliger, *Nature Chemistry*, 2022.
2. A. I. Taylor, V. B. Pinheiro, M. J. Smola, A. S. Morgunov, S. Peak-Chew, C. Cozens, K. M. Weeks, P. Herdewijn and P. Holliger, *Nature*, 2015, **518**, 427-430.
3. H. Hoshino, Y. Kasahara, M. Kuwahara and S. Obika, *J Am Chem Soc*, 2020, **142**, 21530-21537.
4. V. B. Pinheiro, A. I. Taylor, C. Cozens, M. Abramov, M. Renders, S. Zhang, J. C. Chaput, J. Wengel, S.-Y. Peak-Chew, S. H. McLaughlin, P. Herdewijn and P. Holliger, *Science*, 2012, **336**, 341-344.
5. G. Houlihan, S. Arangundy-Franklin, B. T. Porebski, N. Subramanian, A. I. Taylor and P. Holliger, *Nature Chemistry*, 2020, **12**, 683-690.
6. A. I. Taylor and P. Holliger, *Current protocols in chemical biology*, 2018, **10**, e44.
7. A. I. Taylor and P. Holliger, *Nature Protocols*, 2015, **10**, 1625-1642.
8. J. Zhang, K. Kobert, T. Flouri and A. Stamatakis, *Bioinformatics*, 2014, **30**, 614-620.
9. V. Jalili, E. Afgan, Q. Gu, D. Clements, D. Blankenberg, J. Goecks, J. Taylor and A. Nekrutenko, *Nucleic Acids Res*, 2020, **48**, W395-W402.
10. B. Langmead and S. L. Salzberg, *Nature Methods*, 2012, **9**, 357-359.
11. H. Li, *arXiv preprint arXiv:1303.3997*, 2013.
12. G. E. Crooks, G. Hon, J. M. Chandonia and S. E. Brenner, *Genome Res*, 2004, **14**, 1188-1190.
13. S. Arangundy-Franklin, A. I. Taylor, B. T. Porebski, V. Genna, S. Peak-Chew, A. Vaisman, R. Woodgate, M. Orozco and P. Holliger, *Nature Chemistry*, 2019, **11**, 533-542.
14. F. Madeira, Y. M. Park, J. Lee, N. Buso, T. Gur, N. Madhusoodanan, P. Basutkar, A. R. N. Tivey, S. C. Potter, R. D. Finn and R. Lopez, *Nucleic Acids Res*, 2019, **47**, W636-W641.
